# Resting-State Functional Connectivity and Scholastic Performance in Preadolescent Children: A Data-Driven Multivoxel Pattern Analysis (MVPA)

**DOI:** 10.3390/jcm9103198

**Published:** 2020-10-02

**Authors:** Daniel R. Westfall, Sheeba A. Anteraper, Laura Chaddock-Heyman, Eric S. Drollette, Lauren B. Raine, Susan Whitfield-Gabrieli, Arthur F. Kramer, Charles H. Hillman

**Affiliations:** 1 Department of Psychology, Northeastern University, Boston, MA 02115, USA; westfall.d@northeastern.edu (D.R.W.); s.anteraper@northeastern.edu (S.A.A.); l.raine@northeastern.edu (L.B.R.); s.whitfield-gabrieli@northeastern.edu (S.W.-G.); a.kramer@northeastern.edu (A.F.K.); 2 Northeastern University Biomedical Imaging Center, Northeastern University, Boston, MA 02115, USA; 3 Alan and Lorraine Bressler Clinical and Research Program for Autism Spectrum Disorder, Department of Psychiatry, Massachusetts General Hospital, Boston, MA 02114, USA; 4 Beckman Institute, University of Illinois at Urbana-Champaign, Urbana, IL 61801, USA; lchaddo2@illinois.edu; 5 Department of Kinesiology, University of North Carolina at Greensboro, Greensboro, NC 27402, USA; esdrolle@uncg.edu; 6 Department of Physical Therapy, Movement & Rehabilitation Sciences, Northeastern University, Boston, MA 02115, USA

**Keywords:** academic achievement, reading, resting-state networks, functional MRI

## Abstract

Scholastic performance is the key metric by which schools measure student’s academic success, and it is important to understand the neural-correlates associated with greater scholastic performance. This study examines resting-state functional connectivity (RsFc) associated with scholastic performance (reading and mathematics) in preadolescent children (7–9 years) using an unbiased whole-brain connectome-wide multi-voxel pattern analysis (MVPA). MVPA revealed four clusters associated with reading composite score, these clusters were then used for whole-brain seed-based RsFc analysis. However, no such clusters were found for mathematics composite score. Post hoc analysis found robust associations between reading and RsFc dynamics with areas involved with the somatomotor, dorsal attention, ventral attention, limbic, frontoparietal, and default mode networks. These findings indicate that reading ability may be associated with a wide range of RsFc networks. Of particular interest, anticorrelations were observed between the default mode network and the somatomotor, dorsal attention, ventral attention, and frontoparietal networks. Previous research has demonstrated the importance of anticorrelations between the default mode network and frontoparietal network associated with cognition. These results extend the current literature exploring the role of network connectivity in scholastic performance of children.

## 1. Introduction

Scholastic performance is an important indicator of success in school throughout childhood. Performance on academic subject matter such as reading and mathematics, measured via standardized testing has been associated with greater future academic and vocational performance [1,2]. Since the implementation of the No Child Left Behind Act of 2001, schools are under increasing pressure to deliver on academic milestones and rely on high standardized testing performance of their students for funding [3]. As such, understanding the underlying brain correlates of scholastic performance is important for understanding individual differences in scholastic ability as well as brain health.

One approach to understanding the neural processes associated with scholastic performance is to investigate resting-state functional connectivity (RsFc), which refers to temporally correlated low-frequency fluctuations of blood oxygenation level-dependent (BOLD) signal across disparate brain regions at rest, or during non-task related fMRI acquisition [4]. In other words, the study of RsFc networks endeavors to understand communication between brain regions that are functionally similar. Importantly, resting-state refers to an individual at rest, or when not performing any specific task. An abundance of previous research has reported the occurrence of many distinct resting-state networks, some of the most characterized being the default mode network, somatomotor, dorsal attention, ventral attention (salience), frontoparietal (control), and others (for review see [5]). These RsFc networks have been shown to be robust and reliable [6,7,8] and have been associated with typical development [9] and aging [10]. Additionally, disrupted connectivity of these networks have been associated with psychopathology [11] and health-related factors such as obesity [12] and aerobic fitness [13]. Of particular interest, a number of networks sub-serve cognition, such as the default mode and frontoparietal networks [5]. These two networks are anticorrelated such that the default mode network is deactivated and the frontoparietal network is activated during task performance, and vice versa during rest [14]. The strength of this anticorrelation has been associated with variability in cognitive task performance [15,16]. Taken together, the dynamics of these functional connectivity networks are important for understanding brain functioning and brain health.

Few studies have investigated the association of underlying brain network correlates of scholastic performance. In one investigation of RsFc and scholastic performance, Chaddock-Heyman et al. [17] explored default mode, salience, and frontoparietal network regions of interest (ROIs), using an independent component analysis (ICA) approach, in association with a composite measure of scholastic performance (composed of reading, mathematics, and language) and found that greater scholastic performance was associated with increased functional connectivity within the default mode, salience, and frontoparietal networks. A growing body of literature has investigated brain network correlates of reading ability. One such study in preadolescent children found that scores on a word reading test were associated with positive RsFc between a seed located in the precentral gyrus and other motor regions as well as positive connectivity between Broca’s and Wernicke’s areas. In addition, the authors found greater positive RsFc between a seed located in the left fusiform gyrus and the ventral medial prefrontal cortex and the precuneus/posterior cingulate cortex (default mode network) [18]. A more recent study found that better pre-reading ability (children aged 2–5 years) was associated with stronger negative connectivity between reading seed regions and the default mode network and stronger positive connectivity with motor areas, insula, thalamus, and cerebellum [19]. Additionally, a large meta-analysis investigated brain areas associated with numerical judgement and reading using task-based fMRI found that, in children, a large portion of the cortex including the left frontal, temporoparietal, and occipitotemporal regions were activated during reading ability tasks. In comparison, a much smaller set of regions were activated during numerical judgement tasks, including the right inferior frontal gyrus, middle frontal gyrus, left superior frontal gyrus, and left middle occipital gyrus [20]. However, very few studies have focused on RsFc networks and their associations with scholastic measures of reading and math.

Much of the RsFc literature has utilized a priori seed regions and ROI analyses, which can contribute to inconsistencies between studies. Previous research has found that functional connectivity networks can be heterogeneous, for example, different areas of the frontoparietal network are associated differentially with default mode and dorsal attention networks [21]. In addition, these constrained analyses can overlook important connectivity patterns that exist outside of typical networks. As such, the primary objective of the current study was to make use of an agnostic data-driven approach by using multi-voxel pattern analysis (MVPA) to investigate RsFc patterns associated with the scholastic performance measures of reading and mathematics. Additionally, the current analysis used a preprocessing technique, aCompCor, that allows for the interpretation of anticorrelations [22]. Accordingly, we predicted that greater reading and mathematics scores would be associated with greater functional connectivity across a number of regions associated with the default mode, salience, and frontoparietal networks. Further, we predicted that anticorrelations between the default mode and frontoparietal networks would be associated with greater reading and mathematics scores. A secondary hypothesis predicted that because the analyses were not constrained to a particular ROI, increased functional connectivity would be found across larger regions of the cortex.

## 2. Materials and Methods

### 2.1. Participants

One-hundred and ninety-two preadolescent children were recruited from the East-Central Illinois area and completed an MRI scan. Data used for this analysis was taken from the FITKids2 (ClinicalTrials.gov: NCT01334359) clinical trial. Participants were excluded if (i) they did not complete both resting-state blocks (n = 16), (ii) had missing brain slices in the field-of-view (n = 12), or (iii) they had excessive removal of scans after scrubbing resulting in less than 5-min [23] of useable data (n = 67; see ‘MRI Preprocessing’ for criteria). Data from 97 participants were used for final analysis (Table 1). There were no significant differences between participants who were included or excluded from analysis based on age, sex, SES, IQ, reading composite, or math composite (all *p*’s > 0.05). Legal guardians provided written informed consent and participants provided written informed assent in accordance with the Institutional Review Board of the University of Illinois at Champaign-Urbana. Legal guardians completed health history and demographics questionnaires. Based on these questionnaires, participants included in this analysis did not receive special educational services from their school, were free of neurological or physical disabilities, and had normal or corrected-to-normal vision.

### 2.2. SES

Socioeconomic status (SES) was assessed using a trichotomous index based on the following: (1) highest level of education obtained by the mother and father, (2) number of parents who worked full time, and (3) participant in a free or reduced-price lunch program at school [24].

### 2.3. IQ and Scholastic Performance

IQ was assessed using the Brief Intellectual Ability of the Woodcock-Johnson III [25]. Scholastic performance was collected using the Kaufman Test of Educational Achievement, Second Edition KTEA-II, [KTEA-II] [26]. Subtest composite scores for reading (word recognition and reading comprehension) and mathematics (math concepts and applications, and math computation) were collected and age standardized scores (Mean = 100, SD = 15) were calculated based on metrics provided by the KTEA-II. The word recognition task consisted of pronouncing increasingly difficult words and the reading comprehension task was composed of matching words with pictures, acting out the action of words, and answering reading passage questions. The math concepts and applications test involved simple math concepts such as comparing and rounding numbers, algebra, calculus, and trigonometry. The math computation test required calculating answers to addition, subtraction, multiplication, and division problems.

### 2.4. MRI Acquisition

Imaging data were collected on a 3T Siemens Magnetom Trio whole-body scanner with 12-channel radiofrequency head coil (Siemens Healthcare, Erlangen, Germany). High-resolution structural data were acquired using a T1-weighted MPRAGE sequence with 0.9 mm isotropic resolution (TR = 1900 ms, TE = 2.32 ms, TI = 900 ms) over 4 min 26 s. Two resting scans were collected for a total of 8 min using a T2*-weighted EPI sequence (TR = 2000 ms, TE = 25 ms, flip angle = 90°, GRAPPA acceleration factor = 2, 92 × 92 matrix resolution, voxel size 2.6 × 2.6 × 3 mm). Participants were asked to lay still with eyes closed during the resting scans. 

### 2.5. MRI Preprocessing

Data were preprocessed using the default analysis pipeline in CONN toolbox [27], which includes realignment, slice timing correction, outlier detection, segmentation, normalization with respect to MNI template, smoothing (6-mm FWHM kernel). To assure scan quality, the Artifact Detection Toolbox (ART) (http://www.nitrc.org/projects/artifact_detect) was used to flag scans with mean signal intensity outside 3 standard deviations from global mean and/or 0.5 mm scan-to-scan motion, these “invalid scans” were then regressed out. After data scrubbing, a minimum of 5-min scan time was required to include a participant in the analysis [23]. Band pass filtering was executed at 0.008–0.1 Hz. We used a component-based noise correction method (aCompCor) for denoising (Behzadi et al., 2007) as implemented in the CONN toolbox, which produces similar results to global signal regression (GSR; explicit inclusion of gray matter signal in the noise estimation) in terms of removal of motion-related artifacts, without many of the potential downsides, as evaluated by Ciric et al. [28]. The added advantage of aCompCor is that the method allows for interpretation of anticorrelations, which other methods such as GSR do not [29]. Thus, the combination of aCompCor and ART toolboxes, allows for an optimized pre-processing approach for the analysis of functional connectivity data. While the removal of 67 participants due to scrubbing related issues resulting in scans with less than 5-min of useable data is quite large, previous papers have found a 30–50% (in normal and ADHD populations) scan attrition rate due to motion in preadolescent children using even less stringent movement criteria [30]. In addition, head motion artifacts have been found to influence intrinsic functional connectivity measurements [31], as such, care was taken to sufficiently remove scans with motion. Because of the initial sample size, head-motion-related artifacts, as well as the high amounts of motion in a preadolescent population, the authors opted to use the stringent quality control methods mentioned above in the data analysis pipeline. 

### 2.6. Multi-Voxel Pattern Analysis

Whole-brain connectome-wide MVPA was used as an agnostic, data driven approach to identify seed regions for standard seed-to-voxel analysis (CONN toolbox and has been described in previous studies [32,33,34,35]). Principal components analysis (PCA) was used to reduce the dimensionality of the resultant data. First, 64 PCA components were retained for each participant’s voxel-to-voxel correlation structure. A second PCA was run across all participants and the first 5 components were retained to maintain a conservative 20:1 ratio of participants-to-components [32]. An *F*-test was performed on all 5 MVPA components. Scholastic performance measures (reading composite and math composite) were entered separately in the MVPA analysis to determine patterns of functional connectivity associated with each of these measures, for a total of two separate MVPA analyses. IQ and SES were entered as covariates as they both correlated with scholastic performance measures. In addition, mean motion did not correlate with IQ, SES, or scholastic performance measures (all *p*’s > 0.05). A height-level statistical threshold of *p* < 0.001, cluster threshold of *p* < 0.05 false discovery rate (FDR)-corrected, and k > 50 were used to determine significant clusters. These clusters were then used as seeds for seed-to-voxel post hoc RsFc analysis to explore patterns of functional connectivity differences between these seed time-courses and those with the rest of the brain that was associated with scholastic performance measures (reading composite, and math composite). Post hoc analyses used a height threshold of whole-brain *p* < 0.001 and FDR-corrected cluster threshold of *p* < 0.05 with non-parametric (1000 permutations) statistics to reduce Type 1 error due to multiple comparisons [36]. An additional post hoc analysis was conducted by adding mean motion as a covariate and the patterns of connectivity did not appreciably change.

## 3. Results

### 3.1. MVPA Results

**Figure 1:**
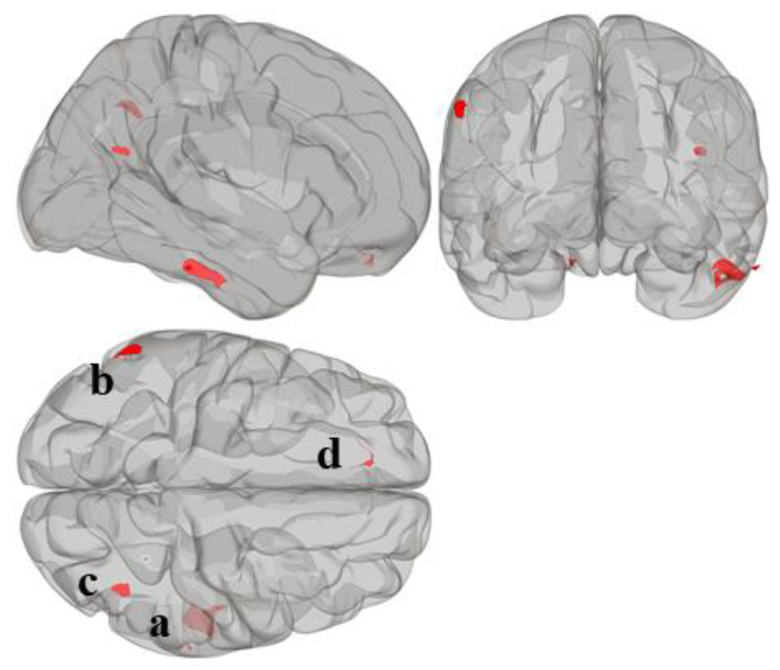
Whole-brain MVPA results indicating the regions that show connectivity patterns significantly associated with reading composite score. MVPA clusters are referred to as (**a**–**d**).

Statistically significant seed regions from the MVPA are displayed in Figure 1 and tabulated in Table 2. A whole-brain height-threshold of *p* < 0.001 and FDR-corrected cluster-threshold of *p* < 0.05 were used to determine significant clusters. MVPA revealed four significant clusters associated with reading composite score located in the right inferior temporal gyrus (cluster a); left supramarginal gyrus (cluster b); right lateral occipital cortex, superior division (cluster c); and left frontal pole (cluster d). There were no significant clusters associated with mathematics composite score.

### 3.2. Post Hoc Seed-to-Voxel Analysis of MVPA-Derived Clusters of Interest

**Figure 2:**
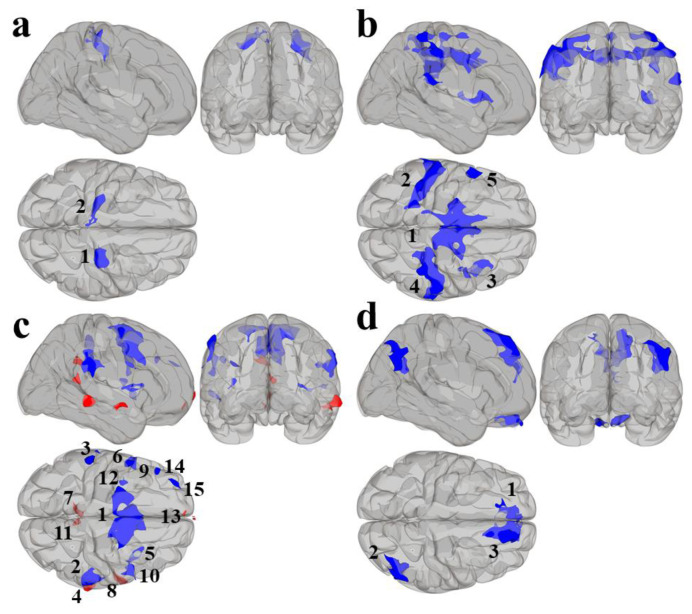
Results from the second-level seed-to-voxel RsFc analysis for MVPA Clusters (**a**–**d**). Red refers to positive associations and blue refers to negative associations. The numbers refer to the specific cluster within each MVPA cluster.

**Figure 3:**
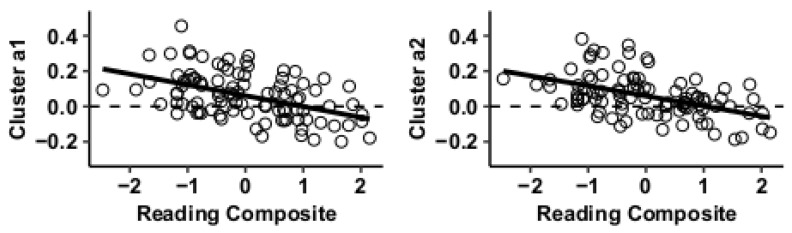
Whole-brain functional connectivity correlations with reading composite score for MVPA cluster a.

**Figure 4:**
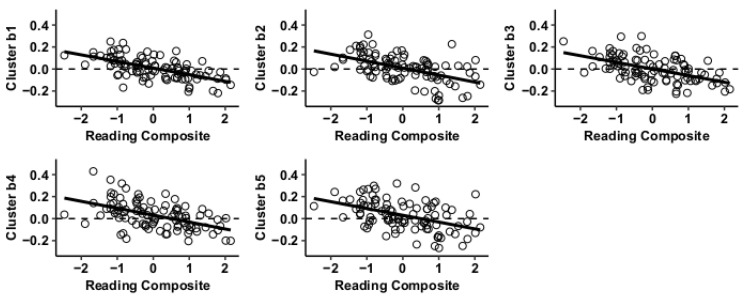
Whole-brain functional connectivity correlations with reading composite score for MVPA Cluster b.

**Figure 5:**
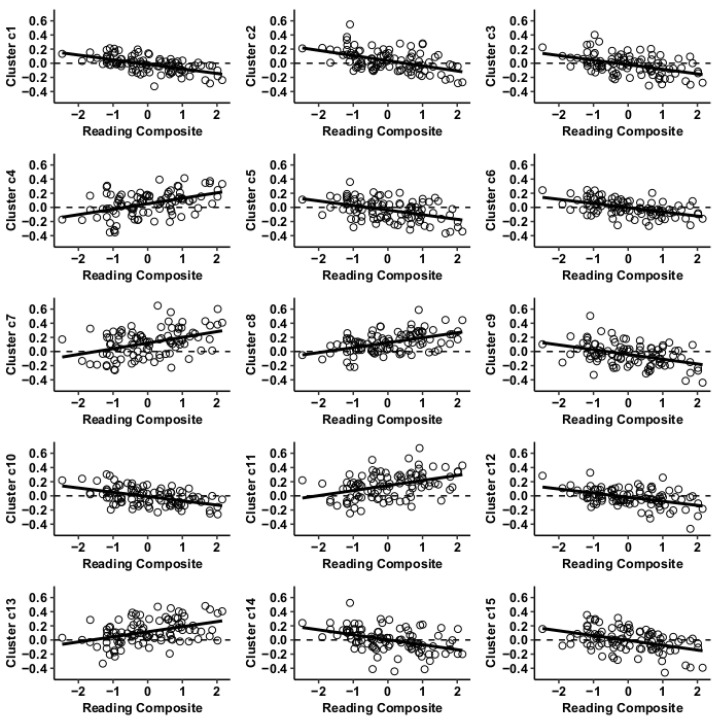
Whole-brain functional connectivity correlations with reading composite score for MVPA Cluster c.

**Figure 6:**
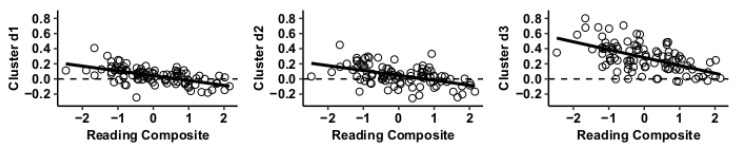
Whole-brain functional connectivity correlations with reading composite score for MVPA Cluster d.

Results using the MVPA-derived clusters are displayed in Figure 2 and tabulated in Table 2. Figure 3, Figure 4, Figure 5 and Figure 6 provide scatterplots of post hoc analysis between RsFc and reading composite score for each MVPA cluster. A height threshold of whole-brain *p* < 0.001 and FDR-corrected cluster threshold of *p* < 0.05 with non-parametric (1000 permutations) was used for post hoc characterization. The seed region located in the right inferior temporal gyrus (cluster a) was found to be anticorrelated with the somatomotor network as a function of reading composite (Figure 3). Moreover, the seed region located in the supramarginal gyrus (cluster b) was found to be anticorrelated with the somatomotor, dorsal attention, ventral attention, frontoparietal, and default mode networks as a function of reading composite (Figure 4). The seed region located in the right lateral occipital cortex, superior division (cluster c) was found to be positively correlated with default mode network, but anti-correlated with somatomotor, dorsal attention, ventral attention, and frontoparietal networks as a function of reading composite (Figure 5). Lastly, the seed region located in the left frontal pole (cluster d) was found to be anticorrelated with limbic, frontoparietal, and default mode networks as a function of reading composite (Figure 6). 

## 4. Discussion

This study utilized a data-driven and unbiased, connectome-wide MVPA approach to examine whole-brain RsFc in association with reading and mathematics composite scores in a population of typically developing 7- to 9-year-old children. Our main findings indicate that a number of network connectivity patterns are associated with performance on a composite measure of reading achievement. More specifically, seed regions in the right inferior temporal gyrus (cluster a) and left frontal pole (cluster d) showed increased anticorrelations between seed regions located in the limbic network and somatomotor, frontoparietal, and default mode networks. Further, these seed regions showed reduced within limbic network connectivity associated with reading composite score. A similar effect was seen for the seed region located in the left supramarginal gyrus (cluster b), which found increased anticorrelations between the seed region located in the default mode network and somatomotor, dorsal attention, ventral attention, and frontoparietal networks. Finally, an interesting pattern of results appeared in relation to the right lateral occipital cortex, superior division (cluster c), which found increased positive correlations with the default mode network, but increased anticorrelations between the seed region located in the dorsal attention and default mode networks with somatomotor, dorsal attention, ventral attention, and frontoparietal networks associated with reading composite score. 

These patterns are similar to those reported by Chaddock-Heyman et al., [17] in that the default mode network shows greater within network connectivity associated with greater reading composite scores. However, the MVPA in this study did not find significant clusters overlapping with frontoparietal and ventral attention (salience) networks. Rather, this analysis found ROIs located in the limbic, default mode, and dorsal attention networks that were associated with reading composite score. In addition, this analysis found that many of the connectivity patterns between these regions and other areas of the brain showed reduced functional connectivity. In particular, there is an anticorrelation within cluster c (dorsal attention and default mode networks) whereby this ROI shows increased connectivity with default mode networks and decreased connectivity with somatomotor, dorsal attention, ventral attention, and frontoparietal networks with greater reading composite score. 

Interestingly, there is a growing body of literature investigating various aspects of reading, reading dysfunction, and functional connectivity networks. For example, one investigation used a Word Reading subtest from the Wechsler Individual Achievement Test—Second Edition WIAT-II, [WIAT-II] [37] and found that, in typically developing children (ages 8–14), word reading was associated with increased functional connectivity between the left precentral gyrus and other motor regions. In addition, the authors found that the connectivity associated with word reading, between left fusiform gyrus and either the left inferior frontal gyrus opercularis (Broca’s area) and the left inferior parietal lobule was negative in children, but positive in adults, suggesting that positive connectivity between these regions was beneficial for word reading in adults, but not children. Finally, the authors found that greater positive RsFc between the left fusiform gyrus and both the ventral medial prefrontal cortex and the precuneus/posterior cingulate cortex (default mode network) was associated with greater word reading in children, whereas in adults this pattern showed greater negative RsFc associated with greater word reading score [18]. The results from this study parallel the increased default mode network connectivity associated with word reading. However, the current study extends these previous findings by demonstrating wide ranging anticorrelations in the somatomotor, dorsal attention, ventral attention, and frontoparietal networks as well.

The identification of associations between reading composite and areas of the brain that sub-serve the dorsal attention, limbic, frontoparietal, and default mode RsFc networks is understandable given the importance of these networks with various cognitive abilities, internal and external attention, as well as the protracted development shown in these networks in preadolescence. Resting-state networks throughout development show a pattern of ‘segregation’ or decrease in functional connectivity between anatomically close regions and ‘integration’ or increase in functional connectivity across selected regions anatomically distant [38]. The interrelated dynamics of the default mode and frontoparietal networks are important for cognitive performance, for example, anticorrelations between these networks are predictive of cognitive performance [15,16]. Further, throughout development, the pattern of connectivity between default mode and frontoparietal networks changes from a positive correlation in children to an anticorrelation in young adults and, after controlling for motion, positive correlations remain below statistical significance in adults [22]. The dorsal attention network is associated with top-down, goal-directed selection of stimuli and responses, whereas the ventral attention (salience) network is specialized for detecting salient events [39]. Dynamic connectivity between the default mode and limbic networks has been associated with simulating events that are associated with achieving a goal. However, also, the limbic network is implicated in a range of emotional processing such as receiving an award or punishment, as well as limbic network dysfunction being associated with mood and anxiety disorders [40]. Finally, the association with reading performance and the somatomotor network is unsurprising given previous findings associating word reading with functional connectivity in motor regions [18]. A lack of findings with mathematics composite score and any MVPA seeds could either be due to a lack of associated functional connectivity patterns with this measure or a lack of power due to the MVPA, whereas an ROI approach may be more powered toward finding an association. 

These findings should be interpreted in light of several limitations. The sample includes a discrete age range of pre-pubescent 7–9-year-old children. Due to the field of view used in the fMRI acquisition, it was not uncommon for scans to obviate the cerebellum, thus this study cannot comment on the role of cerebellar RsFc and scholastic performance scores. In addition, as the data were cross-sectional in nature, causal associations between network connectivity and reading and mathematics composite scores cannot be inferred. Similarly, because RsFc was assessed at one time point, this study is unable to account for fluctuations of attention that may occur over longer periods of time. Additionally, we did not assess test anxiety; however, these tests were conducted in a controlled laboratory environment and had no bearing on participants’ school grades. Given the age group of participants, there was a high amount of movement that occurred during collection resulting in the loss of a number of participants who did not meet the required criteria of at least 5-min of clean scanning data. However, the stringent criteria is also a strength of this investigation, as previous research has found that movement causes issues with RsFc measures [31]. Further, the sample of 97 participants is still quite large compared to other RsFc MVPA studies. Lastly, each individual resting-state scan was collected over 4 min, which may be a short time to allow for network stability, future studies should consider longer scanning times.

## 5. Conclusions

To the best of our knowledge, this is the first data-driven analysis of RsFc and scholastic performance. Using connectome-wide MVPA, we report robust associations between reading and RsFc dynamics with areas involved with the somatomotor, dorsal attention, ventral attention, limbic, frontoparietal, and default mode networks. These findings indicate that reading ability may be associated with a wide range of RsFc networks. Of particular interest, anticorrelations were observed between the default mode network and the somatomotor, dorsal attention, ventral attention, and frontoparietal networks. Overall, these findings advance our understanding of the underlying RsFc networks associated with reading and support the utility of whole-brain unbiased data-driven approaches.

## Figures and Tables

**Table 1:** Participant demographics and scholastic performance.

Measure	Mean
n	97 (53 female)
Age (years)	8.7 ± 0.6 (range: 7.6–9.8)
IQ	108.7 ± 13.0
SES	1.9 ± 0.8
Reading Composite	111.9 ± 13.7
Mathematics Composite	109.5 ± 15.1

**Table 2:** Reading composite functional connectivity.

FC Regions	Peak Coordinates (MNI)	Peak Coordinate Brain Region	BA	Voxels per Cluster (k)							Total k	T-Score
				Visual	Somatomotor	Dorsal Attention	Ventral Attention	Limbic	Frontoparietal	Default		
MVPA a	+52 −34 −28	Inferior temporal gyrus (r)	BA20	0	0	7	0	114	4	0	212	-
Cluster 1	+24 −26 +52	Precentral gyrus (r)	BA3	0	298	0	0	0	0	0	488	−5.12
Cluster 2	−26 −26 +54	Precentral gyrus (l)	BA3	0	259	6	0	0	0	0	372	−5.25
MVPA b	−58 −52 +36	Supramarginal gyrus (l)	BA40	0	0	0	0	0	0	62	80	-
Cluster 1	−12 −04 +42	Juxtapositional Lobule Cortex (l)	BA6	0	1186	660	687	0	100	2	3295	−7.59
Cluster 2	−56 −30 +36	Supramarginal gyrus, anterior division (l)	BA2	0	247	766	301	0	94	1	1556	−5.99
Cluster 3	+38 +16 +04	Insular cortex (r)	BA48	0	43	0	305	0	2	0	368	−6.07
Cluster 4	+60 −34 +20	Planum temporale (r)	BA42	0	16	0	145	0	0	0	183	−5.80
Cluster 5	−52 +08 +36	Precentral gyrus (l)	BA44	0	10	93	0	0	22	25	175	−5.32
MVPA c	+40 −64 +22	Lateral occipital cortex, superior division (r)	BA39	0	0	18	0	0	0	20	53	-
Cluster 1	+10 +04 +64	Superior frontal gyrus (r)	BA6	0	422	365	1103	0	128	8	2352	−7.16
Cluster 2	+60 −30 +28	Supramarginal gyrus, anterior division (r)	BA48	0	16	3	389	0	47	0	464	−5.62
Cluster 3	−56 −34 +56	Supramarginal gyrus, anterior division (l)	BA2	0	10	149	65	0	19	0	253	−5.07
Cluster 4	+72 −34 −10	Middle temporal gyrus, posterior division (r)	BA21	0	0	0	0	0	85	99	219	4.85
Cluster 5	+56 +00 +06	Central opercular cortex (r)	BA48	0	11	0	189	0	0	0	212	−4.82
Cluster 6	−54 +08 +32	Precentral gyrus (l)	BA44	0	26	125	42	0	0	0	200	−6.11
Cluster 7	−02 −40 +30	Cingulate gyrus, posterior division (l)	BA23	0	0	0	0	0	0	137	138	4.32
Cluster 8	+64 +04 −12	Superior temporal gyrus, anterior division (r)	BA21	0	25	0	0	0	0	74	132	5.46
Cluster 9	−56 +10 +00	Inferior frontal gyrus, pars opercularis (l)	BA48	0	15	0	80	0	2	0	130	−4.77
Cluster 10	+48 +08 +28	Precentral gyrus (r)	BA44	0	0	88	2	0	0	0	106	−5.52
Cluster 11	+04 −44 +12	Cingulate gyrus, posterior division (r)	BA29	0	0	0	0	0	0	52	93	4.33
Cluster 12	−34 +02 +12	Insular cortex (l)	BA48	0	2	0	66	0	0	0	90	−5.20
Cluster 13	−02 +60 −12	Frontal pole (l)	BA11	0	0	0	0	8	0	35	89	4.57
Cluster 14	−46 +34 +34	Middle frontal gyrus (l)	BA45	0	0	0	0	0	63	0	86	−4.77
Cluster 15	−36 +48 +30	Frontal pole (l)	BA46	0	0	0	56	0	5	5	77	−4.39
MVPA d	−12 +44 −24	Frontal pole (l)	BA11	0	0	0	0	39	2	0	50	-
Cluster 1	−08 +46 +40	Frontal pole (l)	BA9	0	0	0	0	0	46	1057	1347	−7.01
Cluster 2	+50 −64 +30	Lateral occipital cortex, superior division (r)	BA39	0	0	10	0	0	114	490	688	−5.43
Cluster 3	+08 +48 −22	Frontal medial cortex (r)	BA11	0	0	0	0	375	0	3	532	−6.41
